# A Review of the Intelligent Optimization and Decision in Plastic Forming

**DOI:** 10.3390/ma15197019

**Published:** 2022-10-10

**Authors:** Xuefeng Tang, Zhizhou Wang, Lei Deng, Xinyun Wang, Jinchuan Long, Xin Jiang, Junsong Jin, Juchen Xia

**Affiliations:** State Key Laboratory of Materials Processing and Die & Mould Technology, School of Materials Science and Engineering, Huazhong University of Science and Technology, Wuhan 430074, China

**Keywords:** plastic forming, intelligent algorithm, process optimization, data-driven, digital twin

## Abstract

The plastic forming process involves many influencing factors and has some inevitable disturbance factors, rendering the multi-objective collaborative optimization difficult. With the rapid development of big data and artificial intelligence (AI) technology, intelligent process optimization has become one of the critical technologies for plastic forming. This paper elaborated on the research progress on the intelligent optimization of plastic forming and the data-driven process planning and decision-making system in plastic forming process optimization. The development trend in intelligent optimization of the plastic forming process was researched. This review showed that the intelligent optimization algorithm has great potential in controlling forming quality, microstructure, and performance in plastic forming. It is a general trend to develop an intelligent optimization model of the plastic forming process with high integration, versatility, and high performance. Future research will take the data-driven expert system and digital twin system as the carrier, integrate the optimization algorithm and model, and realize the multi-scale, high-precision, high-efficiency, and real-time optimization of the plastic forming process.

## 1. Introduction

Plastic forming is a manufacturing method that realizes volume transfer and obtains shape, size, and performance that meet the requirements via plastic deformation by simultaneously applying a force field or temperature field [1]. As an essential part of advanced manufacturing technology, plastic forming has significant advantages of excellent forming quality, high forming efficiency, and low material waste. It is an effective method for manufacturing high-performance parts in many fields such as aerospace, transportation, and weaponry [2]. However, plastic forming is a complex process with multi-physical field coupling, multi-factor influence, multi-defect constraints, and triple nonlinearity of materials, geometry, and boundaries. It faces many challenges such as difficulty in forming complex components, high forming cost, long forming cycle, and inevitable disturbance factors [3]. The process design and mold manufacturing rely too much on experience. Improving the accuracy of products requires repeated trial and error, resulting in a long development cycle and high cost. Moreover, many forming defects cannot be traced back, leading to the stagnation of the whole process, which seriously hinders the development of the advanced manufacturing industry. Therefore, it is necessary to optimize the plastic forming process to obtain high-performance components, while improving production efficiency and reducing production costs [4].

The traditional optimization methods mainly include trial and error methods based on experience and orthogonal experimental design based on theory and simulation. The trial-and-error method depends entirely on personal experience, with high uncertainty and non-interpretability. Based on probability theory, mathematical statistics, and practical experience, the orthogonal experimental design uses a standardized orthogonal table to arrange the experimental scheme. It calculates and analyzes the results to quickly find a design method for the optimal experimental scheme [5]. However, orthogonal experimental design does not consider the joint action between factors and requires high experimental accuracy, which is not universal for complex plastic forming process optimization. Simulation has the advantages of good visualization and high precision. Still, the optimization method that entirely depends on numerical simulation calculation has low calculation efficiency and is difficult to converge quickly. It cannot realize the automatic optimization design of the plastic forming process and cannot make intelligent judgments on many problems in the process. Therefore, to shorten the production cycle, improve the forming limit and quality, and realize near-net shape forming, the intelligent optimization of the plastic forming process is imminent, which is also the hotspot and difficulty in plastic forming.

Intelligent optimization is essentially an optimization method that determines optimization strategy and solves the optimal model based on the assumption space, and finally predicts or analyzes new data using the learned optimization model. It has attracted increasing research interest in various fields, such as material design [6,7,8], machining [9,10,11,12], welding [13,14,15,16], and plastic forming [17,18,19,20,21]. Generally, the limited set of input data determines the hypothesis space of the model, the optimization strategy determines the criteria for model selection, and the optimization algorithm is used to solve the optimal model. Therefore, under the given conditions of model and strategy, the performance of the intelligent optimization algorithm determines the quality of the whole optimization scheme. Usually, it hopes that a good algorithm can find the global optimal solution with the fastest rate and has good versatility and stability. In plastic forming, an optimization algorithm can solve complex nonlinear models, carry out multi-dimensional and multi-objective optimization, and balance production efficiency and accuracy. With the rapid development of computer software and hardware, plastic forming optimization design based on an intelligent optimization algorithm has been widely used in many scenarios, such as constitutive parameter identification, forming mechanism analysis, and process parameter optimization.

There are many review articles about intelligent manufacturing, but few focus on intelligent process optimization of plastic forming. Li et al. [4] summarized the research progress of deterministic and uncertain optimization methods and technologies in the design optimization of plastic forming and discussed the challenges and problems required to be solved in the design optimization of plastic forming. In this review, the research progress on the intelligent optimization of plastic forming was summarized in Section 2. Then, the data-driven process planning and decision-making system in the plastic forming process were expounded in Section 3. Finally, a summary analysis and outlook on the challenges of the current intelligent process optimization in plastic forming were provided.

## 2. The State of the Art of Intelligent Optimization in the Plastic Forming Process

Plastic forming must first ensure the required shape and dimension of parts. The forming defects that may be caused by the improper plastic forming process include surface cracks, folds, surface pits, surface bubbles, and orange peel surface. The forming quality can be controlled by adjusting process parameters. However, there are usually many process parameters for plastic forming, and there is usually a coupling relationship between parameters. The selection of main parameters and reasonable optimization methods are the most critical. Secondly, the microstructure and performance must also be optimized to ensure that the parts have good mechanical properties. Finally, it is necessary to optimize the workshop scheduling to reasonably arrange production resources and improve production efficiency for the whole production workshop. This section discusses research progress on the intelligent optimization of plastic forming in terms of parts forming quality, performance, and workshop scheduling according to different optimization objectives. Cloud computing in plastic forming and hybrid physics-informed and data-driven modeling are also introduced.

### 2.1. Optimization of Forming Quality and Performance

**Figure 1:**
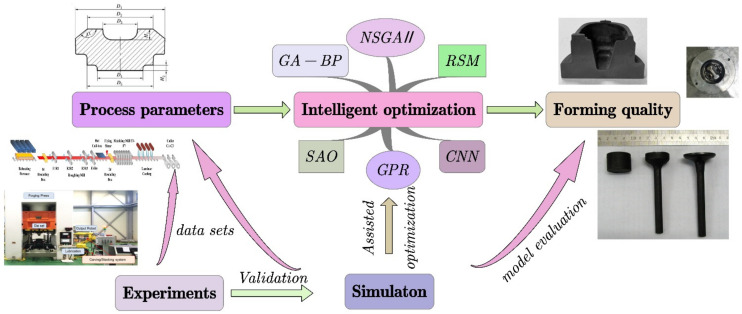
The framework for intelligent optimization of forming quality and performance.

The intelligent optimization of forming quality and performance is mainly carried out by optimizing process parameters. Figure 1 shows the framework for intelligent optimization of forming quality and performance. Geometric parameters of parts and process parameters of the forming process are used as input variables, and forming quality and performance indexes such as forming load, wear degree, springback, grain size, and fatigue strength are used as decision variables. Simulation and experiment are combined in the optimization process to assist optimization, verify model accuracy, and provide original training data. The whole framework aims to accurately establish the relationship between process parameters and forming quality to achieve accurate regulation and efficient optimization.

Plastic forming has many different types of processes, such as stamping, forging, rolling, and spinning, characterized by different forming features and problems. Therefore, it is necessary to formulate the corresponding intelligent optimization scheme for different forming processes to achieve optimal optimization.

#### 2.1.1. Stamping

The primary defects that arise in sheet stamping are cracks, springback, thinning, and wrinkling. These defects are significantly related to blank holder force (BHF), draw-bead geometric parameters, die radius, die gap, and punching speed. Various types of multi-object optimization problems could be generated due to the complex interaction of process parameters. The traditional orthogonal test and Taguchi methods cannot solve all the problems well. Optimizing the stamping process parameter is of great significance in improving the forming quality of stamped parts using intelligent optimization. Liu and Yang [17] optimized the sheet metal forming process using a multi-objective genetic algorithm (MOGA) based on a response surface mode (RSM). Obtaining BHF and draw-bead restrain force as design variables, the fracture, wrinkling, insufficient drawing, and thickness variation of the parts are minimized simultaneously. The proposed method was proven more effective and accurate than the traditional finite element analysis method and trial and error method by an automobile body panel stamping example. Xie et al. [22] proposed a hybrid model based on a restricted Boltzmann machine and a backpropagation neural network. The stamping process parameters of double-C parts were successfully optimized using the improved multi-objective particle swarm optimization (PSO) algorithm with the maximum thinning rate and thickening area as the optimization objectives. Stefanos and Georgios [23] predicted the springback of sheet metal stamping under different input parameters using an artificial neural network (ANN) trained by a Bayesian backpropagation algorithm. Cai et al. [24] compared the four machine learning model of Gaussian process regression (GPR), gradient boosting regression, k-nearest neighbors regression, and multi-layer perception regression on the prediction of maximum thinning and thickening rates, and the gradient boosting regression model had the highest accuracy. This machine learning model provides a fast prediction method for the intelligent optimization of the stamping process.

Many scholars achieved some optimization results using the deterministic optimization scheme, while this optimization may not be stable since the disturbance changes of property of raw material, surface roughness, and forming parameters such as forming temperature, and BHF would give rise to the inevitable quality fluctuation. The internal variability in the forming process should thus be considered. Gantar and Kuzman [25] proposed a method that combines the response surface model (RSM) with the simple stochastic optimization method of Monte Carlo simulation (MCS) to optimize the stamping process from the perspective of stability. The RSM was used to predict the response of the system under a wide range of input variables, and the MCS was used to evaluate the stamping process stability under different blank holder forces. The optimal BHF was finally found to ensure the minimum number of defective products in the production process. Marretta et al. [26] developed a design tool integrating finite element simulation (FES), RSM, and MCS to reduce the part thinning and springback in the stamping of an S-shaped U-channel aluminum part, where BHF was used as the design variable and two noise factors, lubricating conditions and strain-hardening index, were considered. This method can consider process variability effects and provide a precise overview of the possible perturbations the analyzed objective function may undergo.

**Figure 2:**
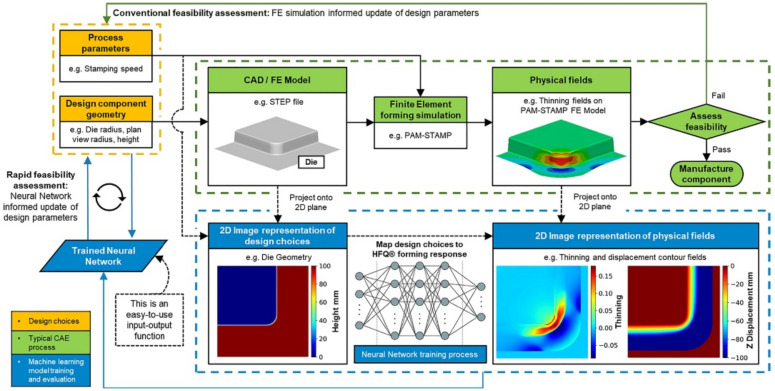
Process optimization of HFQ based on the CNN-based surrogate [27].

The above optimization schemes are all based on scalar methods. When the number of noise factors or design variables to be considered increases, the dimension of the problem usually increases sharply, which will bring a great difficulty to the training and solution of the model and result in low generalization ability. Image-based convolutional neural network (CNN) may effectively handle such problems. Attar et al. [27] proposed a surrogate model based on CNN to optimize the Hot Forming and cold die Quenching (HFQ) process of aluminum alloy. As demonstrated in Figure 2, the CNN extracts geometric features from the design input images, generates corresponding forming response diagrams under HFQ conditions, and predicts the mechanical property parameters such as the maximum thinning rate, thickening rate, and maximum shear angle of the aluminum alloy under the process. It visualizes the stress–strain distribution of the workpiece and guides the early design of the aluminum alloy under the HFQ process. It is worth mentioning that the CNN-based surrogate model can effectively solve the lack of information in the previous scalar-based data model and is applicable to predicting mechanical properties of workpieces under complex processes. Zhou et al. [28] pointed out that the CNN model is more accurate, general, stable, and informative in predicting the results of stamping and forming physics by comparing the CNN surrogate model and the traditional scalar model. Even for small datasets, CNN surrogate models can extract more effective information.

In addition to optimizing the stamping process parameters to obtain stamping parts with excellent forming quality, some scholars have also predicted the microstructure of stamping parts to acquire important information related to mechanical properties. Chokshi et al. [29] proposed an ANN-based phase volume fraction prediction model for hot stamping, which took the heat treatment history, deformation amount, and deformation temperature as inputs and successfully predicted the final hot stamping phase distribution. Hu et al. [30] proposed a method for SEM image recognition of martensite microstructure based on support vector machine (SVM), which can accurately identify the martensite content in hot stamping parts to determine the strength of stamping parts. Thawin et al. [31] used an ANN surrogate model with time-temperature and deformation as inputs to predict the phase volume fraction after hot stamping, replacing the austenite decomposition model in the hot stamping simulation, significantly reducing the phase transformation calculation time.

**Figure 3:**
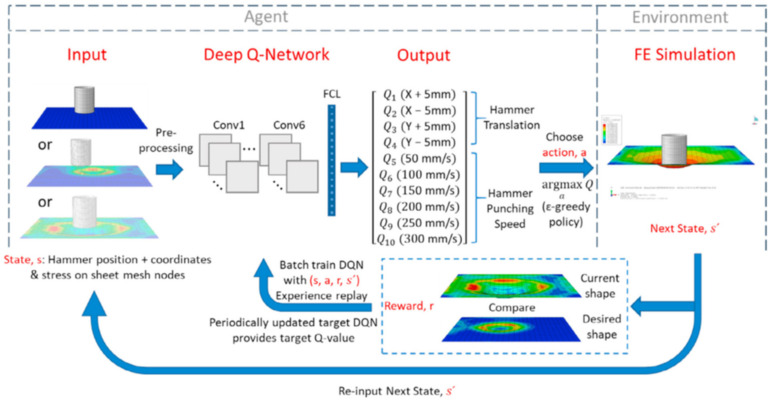
The overall learning algorithm diagram of the RL in free-form stamping of sheet-metals [34].

Scholars usually use surrogate models to establish the response between stamping process parameters and forming goals and then solving the model through different optimization algorithms. This kind of optimization scheme can solve the forming of simple stamping parts under specific conditions. Still, the generalization ability and interpretability are poor, and when optimizing a new stamping process such as HFQ, it is often impossible to optimize due to insufficient empirical data. The CNN model optimizes the stamping process based on images, which can often extract more information from the original data. Previous studies have proved that the surrogate model based on CNN has higher accuracy than the traditional models such as Kriging and radial basis function (RBF) [32,33]. The optimization scheme based on CNN can solve most of the forming defects caused by improper process parameters. However, due to a lack of decision-making ability, the optimization design of stamping dies and the optimization design of the entire stamping process route have not been widely used. It requires a deep combination of CAD, CAE, and intelligent optimization technology and promotes the application of RL and ES in stamping. In the research of Liu et al. [34], the RL algorithm combined with the FES technology was used to simulate the entire stamping process. Finally, the optimal forming path to achieve the target shape was predicted. The overall learning algorithm diagram is shown in Figure 3. The input is the hammer coordinates, the coordinates of each grid node, and von Mises stress derived from simulation, which are transferred into the deep Q-network (DQN) after preprocess. The output is the predicted Q value of each action and then selecting the action with the largest Q value based on the ε-greedy strategy. The corresponding simulation was performed according to the selected action to reach the next state and compare the current shape with the target. If the error is within the specified range, set the reward *r* = 100, and continue the decision of the next action after updating the DQN network in small batches. Otherwise, set the reward *r* = −1, and restart the decision-making with the current state as the initial state. This algorithm realizes the intelligence of the traditional free-form sheet metal stamping process without prior professional knowledge guidance. RL is also applied to directly output the optimal process parameters according to the target shape of the stamping part, which brings great convenience to the early design of the stamping process. Recently, some scholars have used RL to optimize the manufacturing process parameters of a component with variable shapes. Unlike the classical optimization based on the surrogate model and CNN model, this method trains a function that directly evaluates the optimal process parameters (output) with the workpiece geometric structure as the input. It combines RL with surrogate-assisted optimization (SAO) to effectively explore the implicit relationship among process parameters, geometric parameters, and forming quality [35]. In addition, the development of transfer learning has been proven to be an optional strategy to improve the model’s applicability. In the future, the combination of RL and transfer learning will promote the development of process parameter optimization [36].

Table 1 summaries the main published intelligent optimization methods for stamping. The size of dataset, optimization parameters, input parameters, and prediction accuracy are listed for easy comparison.

#### 2.1.2. Forging

Forging can produce structural parts with complex shapes and excellent mechanical properties and is a critical forming technology for large structural components in automobiles, aerospace, and national defense. However, it is difficult to gain an accurate shape with tailored microstructures due to the complex deformation and microstructural evolution behavior. Meanwhile, the forging process involves many disturbances such as multi-field coupling, fluctuations in ambient temperature, and complex heat transfer between the workpiece and the die, making it an arduous task to control the process-structure-property relationships of the forging process. It also reduces the life of the forging die and equipment when an inappropriate process is adopted. Traditional optimization mainly relies on empirical trial and error and finite element analysis. Such methods are very blind and time-consuming, often resulting in a severe waste of materials and equipment [41]. Therefore, the intelligent optimization of the forging process is of great significance for forming high-quality complex forgings and improving the service life of forging dies.

Pre-forging design is one of the important optimization problems in forging. A good pre-forging shape can obtain high-quality forgings, reduce forming resistance, and improve the service life of molds and equipment. Zhang et al. [42] established a surrogate model to predict the relationship between the dimensions of the preform and forming force and maximum die stress in the final forging process using a back propagation neural network (BP). Combing the BP surrogate and GA, the optimal shape and dimensions of preform were obtained to minimize the forging force and die stress. The GA-BP method is an effective approach to optimizing the preforms and shortening the design cycle. It can also be used to optimize the die shape. The GA-BP algorithm is frequently used in size optimization because of its good global optimization ability and high robustness. However, for complex pre-forgings and die shape, the number of involved parameters will rise sharply, leading to the difficulty in collecting enough sample data for training. Torabi et al. [43] optimized the blade pre-forging shape by coupling the RSM with the NSGA-II multi-objective optimization algorithm. In this optimization, the blade shape parameters were set as input variables, and the filling rate, strain distribution variance, flash area, and forging load were the optimization objectives. RSM can quickly establish models and is an effective method to deal with data with errors. However, for high-dimensional problems, the fitting accuracy is poor, the ANN can be considered to replace RSM to build prediction models in this scenario. Wu et al. [44] used the approximate surrogate model to optimize the shape of the pre-forging die of multi-station high-speed forging. They adopted the LHS method to obtain the initial sample points and used the finite element simulation to obtain the response values. Based on the Kriging model and BP neural network, the surrogate model approximates the relationship between the pre-forging station die shape and the forming load and forming quality (with or without folding, filling rate). The GA based on the penalty function is combined to obtain the optimal solution. This study verified the possibility and superiority of the combination of intelligent optimization algorithm and surrogate model and is of practical value and guiding significance for actual production.

**Figure 4:**
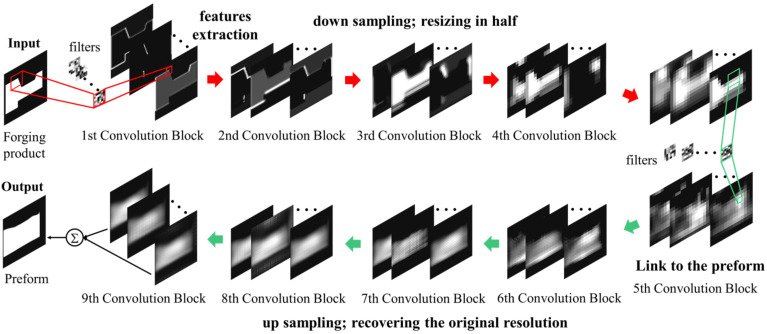
The preform design step during the implementation of the CNN algorithm [18].

The above optimization case shows that the surrogate model is widely used in the pre-forging design. Most researchers have improved the accuracy of the model by using intelligent optimization algorithms. However, process optimization must consider the interpretability of the model and improve its applicability, which has brought challenges to the SAO with its “black box” nature. It is expected to obtain the most accurate prediction model with minor process parameters while ensuring the running speed of the model. Similar to the stamping process above, the intelligent optimization scheme based on CNN has also been applied in pre-forging design. Lee et al. [18] proposed a new method for the preform design of metal forgings based on CNN, as shown in Figure 4. The CNN model is composed of nine convolutional blocks with two convolutional layers and eight pooling layers. It extracted the geometric features of the target forged products and linked them to the corresponding preform shapes. Multiple 3D preform design candidates for one inputted forging product geometry were automatically generated with minimum forging load and without forming defects after training. Then, the best one could be selected depending on the design requirements and the results of the forging simulation for the individual preform candidates. Although this method solves the problem of many shape parameters, it also needs a large number of forging image information for training. It only takes the forging load as the objective to optimize, does not consider the influence of other forming factors, and can only be designed for relatively simple axisymmetric forgings.

**Figure 5:**
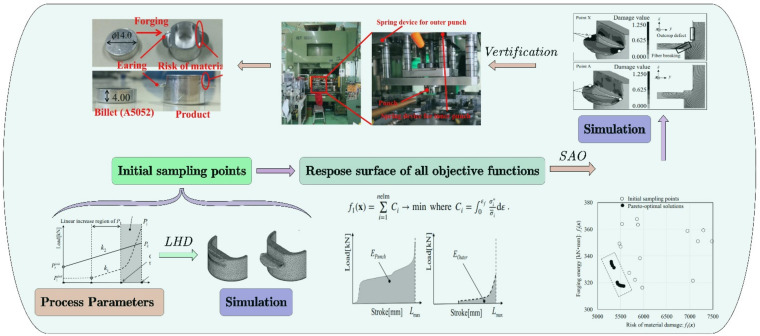
Minimizing risk of crack and forging energy based on a radial basis function-based SAO method [45].

In addition to pre-forging design, there is also substantial research on the optimization of process parameters. Kitayama et al. [45] adopted an RFB-based SAO method to minimize the product damage risk and forging load of the aluminum cold forging process, as shown in Figure 5. The ratio of external punch load, internal punch load, spring stiffness, and the maximum sliding speed of the punch was obtained as input variables that need to be optimized. It successfully reduced the damage risk at the forging ear and the total forging energy, and obtained the streamline along the product shape. The experimental results show that the SAO method based on RBF can replace the complex finite element simulation by establishing a simple approximate model, effectively reducing the simulation calculation cost and improving the optimization design efficiency [46]. However, this study applies the weighted norm to the multi-objective function to search the Pareto frontier. The selection of the weight coefficient is random, which has a certain impact on the optimal solution set. Xu [47] used the multi-objective fuzzy method to optimize the radial forging process of rectangular section work-piece. The radial forging passes, reduction rate, and feed rate were taken as optimization parameters. The optimization objective was a multi-objective weighting function, including forging load, forging permeability, and forging efficiency. The weight selection between multiple objectives depends on personal preference, which is fuzzy. The fuzzy set theory is easy to describe and use people’s experience and knowledge, and thus has the logical reasoning ability that other algorithms are inferior to [48]. Therefore, using the fuzzy method can effectively solve the problems of multi-objective weight combination imbalance and multi-objective function leakage.

It can be seen from the above research that in the process of optimizing the forming quality, there are many variables in the model. Although the accuracy of the model is improved through various optimization algorithms, the interpretability of the model becomes poor, and the model with more variables will generally be less robust, which brings great constraints to the actual production and application [49].

**Figure 6:**
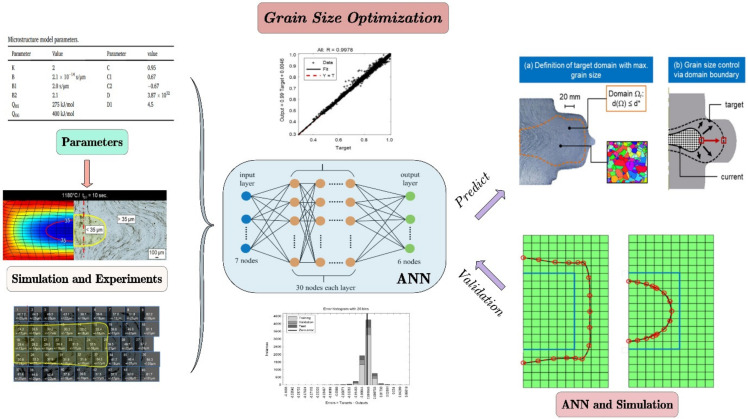
Property control in multi-stage hot forming based on machine learning [19].

Since the internal microstructure of parts usually cannot be measured directly, microstructure modeling is the important issue to solve in intelligent optimization. Bambach et al. [19] proposed that in closed die forging, the microstructure and performance during forging can be directly controlled by adjusting the stroke of the screw press and the dwell time in multi-pass forming. As shown in Figure 6, they defined a region γ, in which all the grain size is smaller than the target value. The evolution of microstructure can be observed by tracking the domain boundary rather than grain size. An ANN model was established to predict the coordinates of the featured node on the edge. The inputs are the temperature, blank transport time, indenter dwell time, and strain rate in the multi-pass forming process. The ANN was trained by the data from finite element simulation based on a grain size model. Therefore, given the initial forging temperature and transport time, it is possible to estimate the position of the grain size boundary based on simulation. Still, the surrogate model may need more training data to improve its accuracy. Some researchers optimize the microstructure through direct grain size measurement or finite element simulation. The workload is large, and the accuracy is difficult to guarantee.

The soft sensing method or material model can estimate microstructure information, such as recrystallization volume fraction (RVG) and average grain size (AGS). The material microstructural model can accurately describe the evolution mechanism of material microstructure and facilitate the optimization of process parameters to control microstructure [50,51]. Chen et al. [52] proposed a microstructure control strategy for the hot forging process of nickel-base superalloy based on PSO. The RVG and AGS were used as the optimization objectives in the control strategy, and PSO was used to optimize the strain rate and forging temperature. Finally, the microstructure with fine grain size and full recrystallization was successfully obtained. This strategy can stably control the microstructure by optimizing the process parameters of high temperature deformation, which provides experience for the future microstructure optimization. At the same time, the strategy can be easily extended to other materials by updating the material model.

In addition to this soft sensor method, it is also possible to predict the properties of the component directly using the metallographic images after forging. Emmanouil et al. [53] proposed a deep learning method to predict materials’ properties directly from the microstructure images. Single regression, fully connected neural networks (FCNNs), and multiple regression CNN were designed and trained. Using the material microstructure images as input, five mechanical properties were predicted, and the accuracy of CNN was 99%. The authors also explained the CNN model, which is usually regarded as a “black box”, to illustrate the ability of CNN to detect relevant microstructure features.

Table 2 summarizes the primary optimization method, optimization parameters, and input parameters in forging. The intelligent optimization methods for pre-forging design are mainly divided into two categories based on scalar models and CNN models. The former is easy to operate but has poor generalization and interpretability. The latter has a good visualization effect, and the optimization speed is faster and more accurate. However, it is currently only suitable for optimizing pre-forgings with simple shapes. For pre-forgings of the same complexity, the CNN model shows more prominent advantages than the scalar model; but as the complexity increases, the number of features that the CNN model needs to extract will also increase, which requires sufficient training data for optimization. However, neither experiment, simulation, nor surrogate model seems to provide a large amount of effective data in a short time, which also shows that data are the core of intelligent optimization. For the control of microstructure and performance, scholars only analyze and optimize the forging results, but cannot realize real-time control.

#### 2.1.3. Rolling

**Figure 7:**
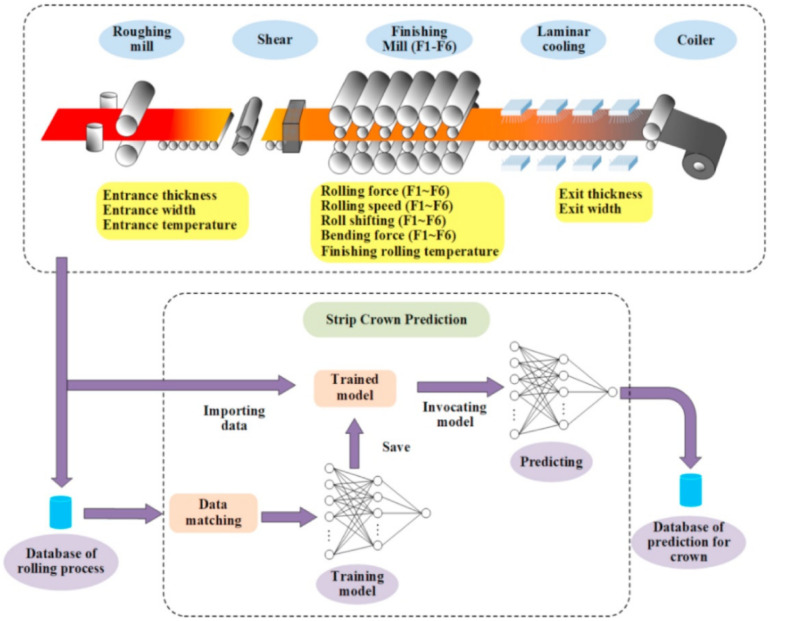
Strip crown prediction process [59].

The rolling process includes many variables, such as temperature, rolling reduction rate, rolling speed, and rolling force. The change of each process variable may have different degrees of influence on the forming quality. The traditional optimization method mainly calculates relevant parameters through dynamic planning combined with finite element analysis. Still, the accuracy and reliability of FES largely depend on human factors, such as constructing mathematical models and setting constraints. The optimization is inefficient since only one solution is obtained at a time using the finite element simulation. Rolling is easier to realize intelligent optimization and control than forging and stamping due to its incremental deformation nature. Wang et al. [58] established a regression model between rolling process parameters and rolling force using a data-driven extreme learning machine (ELM), and the PSO algorithm was used to optimize the model. They compared the prediction results of PSO-ELM with ELM and PSO-SVM. They showed that the prediction accuracy and generalization ability of the PSO-ELM model are better than other models, and it is very suitable for parameter prediction and model optimization of the strip rolling process. Deng et al. [59] applied an ANN-based method to predict the crown of the hot-rolled strip, as shown in Figure 7. The inputs of the ANN model contain 34 process parameters, and 10,133 groups of data acquired from a hot rolling process were used to train the model. A comparison study found that the DNN model has higher prediction accuracy and better generalization ability. Xie et al. [20] developed an online prediction system of mechanical properties of hot rolled steel plate by DNN model, which was trained by 11,101 groups of real-word data. The prediction accuracy of different machine learning algorithms was compared in Table 3. The DNN model yielded the highest prediction accuracy, indicating the great ability to handle large datasets with high dimensional data.

The above prediction model could not capture the influence of noise factors in the production process since it does not consider the impact and genetic dependence of the dynamic changes in the working conditions. Agarwal et al. [60] developed a hybrid model which uses the mill setting and the real-time plant data such as chemical composition, forces, and temperatures and integrates them into a Bayesian format to predict the desired quality attributes as well as microstructural features. This information is combined into Bayesian hierarchical models to create an online tool that predicts the properties of each individual rolled coil, as well as provides information on the batch-to-batch and heat-to-heat variations. The prediction results agreed well with the legacy plant data. This shows that combining the prediction and statistical model, the hybrid model has significant advantages in dealing with optimization problems with noise factors.

**Figure 8:**
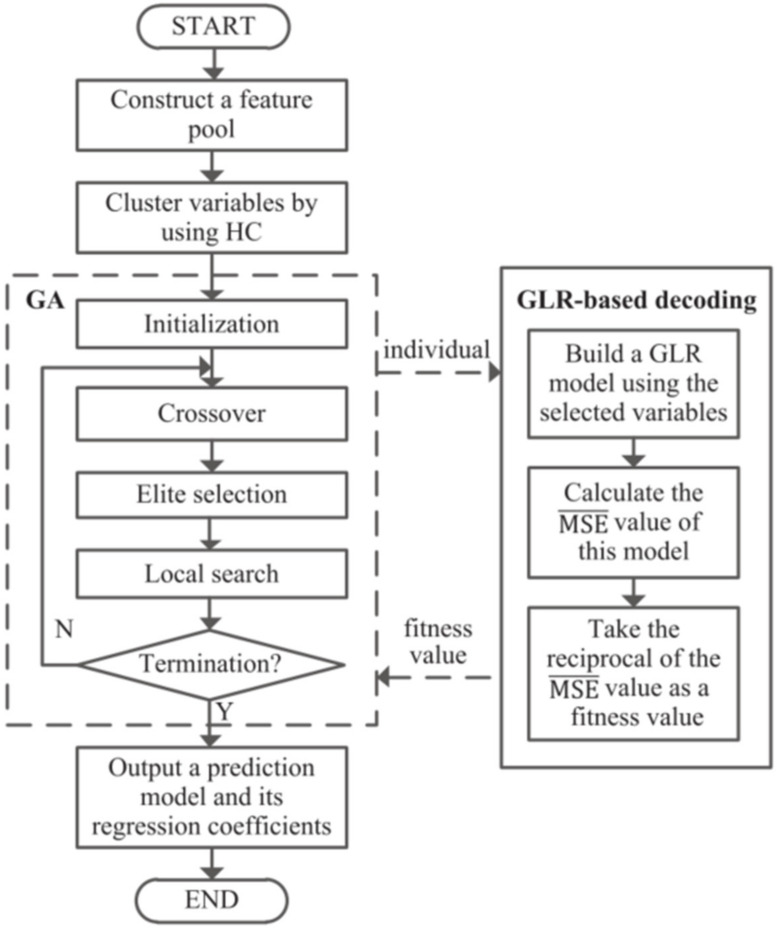
Flowchart of machine-learning and genetic-algorithm-based hybrid method [61].

Both single and hybrid models focus on the predictive accuracy of the model and ignore interpretability. Although the accuracy of the model is improved through various optimization algorithms, the interpretability of the model becomes poor, and the model with more variables will generally be less robust, which brings great constraints to the actual production and application [49]. Ji et al. [61] proposed a hybrid method based on machine learning and GA to obtain the prediction model of strip width deviation after hot rolling. The model can consider both prediction accuracy and interpretability. The flowchart of this method is shown in Figure 8. Firstly, it collects some process variables in the hot rolling process and includes them and some artificially constructed variables in the feature pool. Then, a GA selects a representative variable from each category to form a chromosome, and the individual fitness value is calculated by generalized linear regression. Finally, the optimal model is output by iteration. This method integrates hierarchical clustering, GA, and a generalized linear regression model. It predicted the influence of different process parameters on hot rolling width deviation. At the same time, it makes the model transparent and has great practical application potential. The disadvantage is that the operation speed needs to be improved. Table 4 lists the primary optimization method for rolling.

#### 2.1.4. Spinning

The spinning process has the advantages of low forming load, simple processing technology, and near-net shape and is widely used in the production of axisymmetric parts and structures in aviation, automobile, and other industries [64]. In the spinning process, the spinning roller plays a vital role in forming the part, which affects the forming quality of the spinning part and the evolution of the workpiece shape. However, the roller path is usually an arbitrary curve with high flexibility and diversity, and it is difficult to characterize it clearly with several variables. Meanwhile, changes in the shape of the workpiece will lead to changes in the flange stability and the force state of the forming zone, eventually leading to wrinkling and fracture on the surface of the workpiece. The wheel path and workpiece shape changes, which change over time, making it very difficult to optimize the spinning process [65].

**Figure 9:**
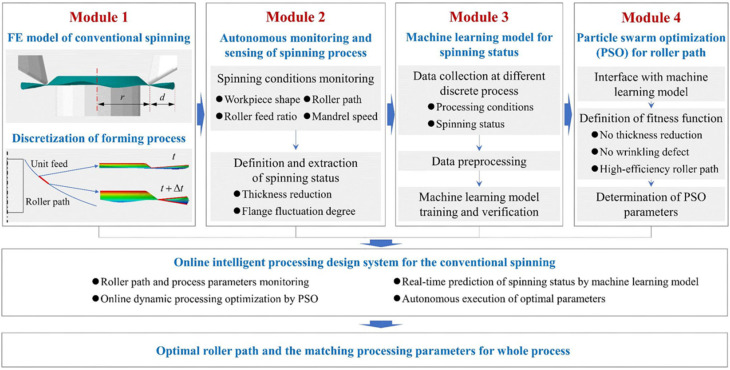
The architecture of the online intelligent design system for the roller path [66].

Gondo et al. [21] proposed a data-driven metal spinning process to generate tool paths to obtain desired dimensions without preparing big data. In addition to the forward prediction, this data-driven process enables the inverse optimization of tool path parameters iteratively by the steepest method using a Jacobian submatrix. Gao et al. [66] proposed an online intelligent optimization system for roll trajectory, as shown in Figure 9. This system is divided into four modules: module 1 is the finite element model of the spinning process, which simulates the entire spinning process and provides a basic platform for intelligent optimization; module 2 monitors and identifies the spinning process state, and uses the continuously collected data for dynamic modeling of the spinning state; module 3 establishes a prediction model between spinning process parameters and states; module 4 uses the PSO algorithm to optimize the wheel trajectory online. Finally, modules 2, 3, and 4 are integrated into the finite element model of module 1 to realize the online intelligent design system of the rotary roller path. The system realizes the functions of spinning state monitoring, real-time prediction of spinning state, online dynamic machining optimization, and autonomous execution of the optimized process. The experimental verification shows that this method can effectively realize intelligent machining optimization and adaptive control of the spinning process. However, this online optimization system is based on virtual finite element simulation and does not actually control the spinning machine. The primary published optimization method for spinning is listed in Table 5.

### 2.2. Workshop Scheduling Optimization

**Figure 10:**
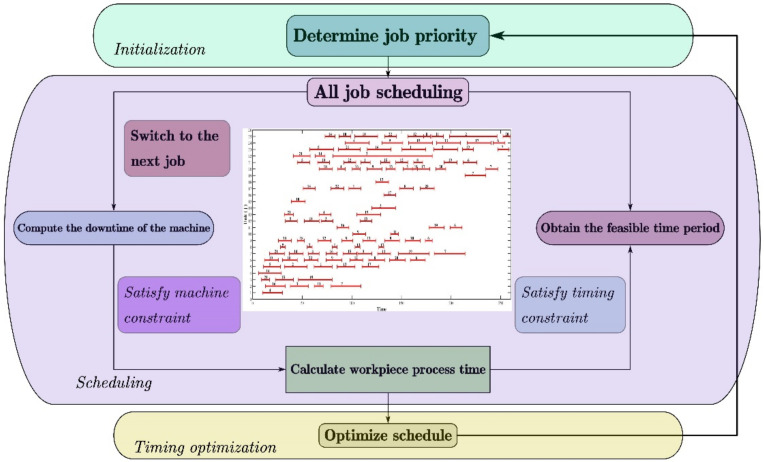
Schematic diagram for workshop scheduling optimization.

The optimization of workshop scheduling in the plastic forming process optimization is also an important problem. Workshop scheduling plays an essential role in the production system by reasonably arranging production resources to shorten production time and improve resource utilization. Figure 10 shows the typical schematic diagram for workshop scheduling optimization. Firstly, the job priority is initialized, and the corresponding machine is determined. Then, the time scheduling of a specific job is carried out, and the feasible operation time of each job is obtained by calculating the downtime of the machine and the processing time of the workpiece. The machine constraints and time constraints are met simultaneously until the scheduling of all jobs is completed. Finally, the total processing time is minimized while the production efficiency is fully guaranteed by optimizing the scheduling planning. The goal of solving the workshop scheduling problem is to determine the workpiece processing sequence of each machine and the specific start time of each process to minimize the total processing time. Wherein, the objective function *L* of the whole optimization, can be expressed as [68]:

(1) $$L=\min\left(\underset{1<i<n}{\max\;C_{i}}\right)$$

where $C_{i}$ is the total time spent by the workpiece.

There are few cases that conform to the workshop scheduling model in actual production, mainly including flow shop scheduling problem (FSP) and flexible job scheduling problem (FJSP). FSP stipulates that each workpiece has the same processing route. FJSP allows multiple machines to be selected for a process, and the processing time on different machines may be different. Both of them are nondeterministic polynomial hard problems [69]. Metaheuristic algorithm is an important method to solve this problem, such as GA, PSO, AI, and ant colony algorithm (ACA). Among them, GA has become one of the most popular algorithms to solve FJSP because of its simple operation, strong universality, and good robustness [70,71].

Seng et al. [72] proposed a low-carbon scheduling method of flexible workshop based on the improved NSGA-II, which reduced the idle time and total energy consumption of the production line through automatic management. Wang et al. [73] proposed a flexible job shop scheduling problem considering preventive maintenance activities and the transportation process. A multi-objective flexible job shop scheduling model integrating GA and DE was established to optimize the total energy consumption and total make span. However, metaheuristics have two main disadvantages. First, because the optimization algorithm has a large amount of computation and cannot be parameterized by a pre-training model, each optimization needs to start from scratch, resulting in a long time response. In addition, the generalization of meta-heuristic algorithm is poor, and it often needs different parameter adjustments for different scheduling problems. So, it is difficult to realize the direct transfer of the algorithm. In recent years, RL, as an important AI technology, has been widely used in robot control, game competition, and other fields [74]. RL is a data-driven method that can learn stable strategies interactively with the environment without labels. Nazari et al. [75] proposed an end-to-end framework to solve the vehicle distribution problem using RL and applied the strategy gradient algorithm to optimize the model parameters, proving the effectiveness of the solution on the medium-sized problem. However, in the job shop scheduling problem, each job has multi-dimensional dynamic characteristics, which is difficult to deal with by RL.

With the development of AI, DRL, which combines the ability of DL to extract high-dimensional features and the ability of RL decision-making learning, has attracted the attention of researchers. It can deal with decision-making problems in high-dimensional state space and high-dimensional action space and mine the characteristics of the problem independently. It uses only the original input instead of artificial features to generate output to realize end-to-end learning. It has been widely used in games, robots, automatic driving, and dialogue systems. Various practical problems have been successfully solved [76,77]. Luo et al. [78] established a workshop scheduling model based on a pointer network and proposed a DRL-based workshop scheduling algorithm. The experimental results on different scale public data sets and production data sets show that the proposed DRL algorithm can achieve better performance. A deep *Q* network (DQN) was developed by Luo et al. [79] to solve the dynamic flexible workshop scheduling problem with random task insertion. The results confirm the superiority and versatility of DQN in dealing with high-dimensional state space and high-dimensional action space.

In summary, various intelligent optimization algorithms can be used in job shopping scheduling optimization. Compared with the optimization of forming quality microstructures in plastic forming, workshop scheduling optimization is more general and can be applied to various workshops. DRL can solve some problems and meet some primary goals. However, there are still problems in the interpretability, convergence, and reusability of the model, so it has not been widely used in actual production. In the future, green flexible workshop scheduling under intelligent manufacturing could be studied in combination with the current development trend of intelligent manufacturing and concepts such as big data, digital twins, the internet of things, and cloud computing. Conducting real-time monitoring of workshop conditions and using intelligent equipment to record carbon emissions and energy consumption during product processing and transportation are needed. The data obtained through supervision are of great research value. Only accurate underlying data can ensure the feasibility and robustness of scheduling [80].

### 2.3. Cloud Computing for Optimization of Plastic Forming

Data are the core of intelligent optimization. With the development of computers and industrial production, many companies have accumulated vast amounts of data. However, due to the different data flows and complex data types, data analysis and calculation face great difficulties, which affect not only users’ acquisition of effective information, but also the economic benefits and future development of enterprises. Therefore, cloud computing technology and AI technology were born to deal with the analysis and calculation of massive data. Cloud computing refers to the computing of data information by utilizing virtualized resources. Compared with ordinary computers, its computing power has been dramatically improved. Cloud computing divides the computing process into several parts, which are finally integrated and stored in the cloud to support the efficient processing of data information [81,82]. Because cloud computing has the advantages of self-service, resource sharing, rapid response, and metering service, it can effectively solve the problems of slow response, low generalization, and difficult quantification of control in intelligent optimization of plastic processes. It has been attempted to be applied to intelligent optimization of plastic forming processes.

**Figure 11:**
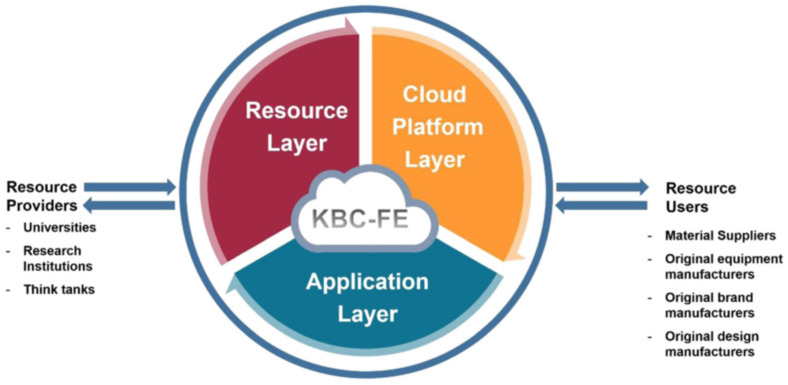
The frame work of KBC-FE [85].

Wang et al. [83] developed a knowledge-based cloud simulation platform (the knowledge-based cloud FE (KBC-FEA)). Cloud simulation was performed on the hot stamping forming process of AA6082 aluminum alloy dome-shaped parts under different conditions, and the temperature and strain rate windows of the part during the hot stamping process were determined. The knowledge-based cloud simulation platform allows multi-objective finite element simulation in the cloud computing environment, which effectively shortens the calculation time and fills the defect of the high time cost of finite element simulation. Wang et al. [84] further developed the platform, as shown in Figure 11. The framework consists of a resource layer (knowledge layer), a cloud platform layer, and an application layer. The resource layer is used to provide data, and the cloud platform layer virtualizes knowledge resources through a unified transformation technology and saves them in the cloud in the form of functional modules for users to access. The cloud platform layer is the control center of KBC-FE, responsible for resource identification, connection, matching, monitoring, and support. The application layer provides man-machine interface, and realizes multi-object simulation by executing multiple function modules on the platform at the same time. The author applied the platform to the optimization of hot stamping forming process of AA6082 aluminum alloy U-shaped parts and used the developed functional modules to predict the forming ability, quenching efficiency of the tools, and strength after forming of the parts. Luan et al. [84] developed an intelligent optimization platform for guiding the aluminum alloy warm/hot stamping process based on cloud computing. The platform consists of two modules, “Tailor” and “Uni-Form”. The Tailor module is used for computing an optimal processing route of aluminum alloy sheet under given conditions, such as forming temperature, forming speed, transfer time, and artificial aging parameters. The Uni-Form module verifies and re-optimizes the processing route optimized by the Tailor module, and automatically configures the production line through the internet to automatically form parts. This two modules are integrated in the SMARTFORMING platform, an online platform that realizes the virtualization and streamlined optimization of sheet metal forming processes, and improves the experimental accuracy and greatly reduces the workload. The data-based cloud computing technology is mainly combined with finite element software. Rapid simulation is realized by developing a cloud platform suitable for specific processes, which expands the functions of finite element software and reduces the time cost for the rapid and accurate establishment of material models.

### 2.4. Hybrid Physics-Informed and Data-Driven Modeling

Over the past decades, trial-and-error experiments and physics-informed modeling have been widely employed to optimize the plastic forming processes. However, the trial-and-error method is time-consuming and has a high expense of experimental tests because of many processes and material parameters [70]. Physics-informed modeling methods are powerful tools to reveal complex mechanisms during forming processes, especially numerical simulation, but the simulation efficiency and accuracy are significantly influenced by several factors [71]. First, extremely fine mesh structures, essential to accurately simulate intricate deformation behaviors, can result in high computational demand. Second, since there are multi-scale and multiphysics natures of forming processes as well as complex boundary conditions, it is difficult to properly formulate the governing equations and solver settings. Third, the processing of experimental data for calibration and validation requires many manual operations, and its integration with simulation remains unsolved. Therefore, it is significant to develop an alternative intelligent optimization approach in plastic forming.

It is worth noting that the data-driven method has emerged as a promising alternative due to its enhanced data accessibility and parallel computing, but it relies on a substantial amount of labeled high-fidelity data, which are difficult to obtain by experiments or simulations [72]. This greatly impedes the wide application of the data-driven method. As described in Section 2.1 and Section 2.2, the data-driven machine learning method is increasingly used to predict various objects, including deformation behavior and service life during plastic forming. When it comes to complex problems such as springback, although the data-driven machine learning method has higher prediction accuracy than the physics-informed modeling, the entire physics of processes are modeled without understanding the process, which easily leads to poor generalization ability and interpretability. Thus, it could predict completely wrong results when the parameters go beyond those used to train the model [73]. In fact, the prediction accuracy and generalization ability of the data-driven model depend on the size of the data set and the structure of the model. When the training data set is large enough, the machine learning model exhibits good predictability and generalization ability. However, such requirement of big data for training the neural networks is not always available for scientific problems [74].

The hybrid physics-informed and data-driven machine learning model has been developed to integrate the advantages of the data-driven method, classical physics-informed modeling and experimental data. Zhang et al. [75] proposed a physics-informed deep learning framework for metamodeling of nonlinear structural systems with scarce data, in which the embedded physics can alleviate overfitting issues, reduce the need for big training datasets, and improve the robustness of the trained model for more reliable prediction, and thus exhibits better performance than classical non-physics-guided data-driven neural networks. Haghighat et al. [76] presented a novel framework for constitutive model characterization and discovery based on the physics-informed neural networks (PINN) by embedding complex inequality constraints of elastoplasticity theory in the PINN loss functions and found that the framework can efficiently and accurately recover the underlying constitutive models on a wide range of material parameters and stress–strain curves. Jiang et al. [77] developed the energy-based physically informed deep neural network by incorporating surface-elasticity effects based on the Gurtin-Murdoch interface model, and results show that this approach is capable of predicting the size-dependent displacement and stress fields of nanoporous aluminum, with a high degree of correlation with the exact solutions and finite-element results. Vlassis et al. [78] introduced a deep learning framework designed to train smoothed elastoplasticity models with interpretable components, including the stored elastic energy function, yield surface, and plastic flow; the Leveraging Sobolev training was adopted to control the derivatives of the learned functions, and results show that the obtained machine learning elastoplasticity models with excellent learning capacity are thermodynamically consistent and interpretable. Koeppe et al. [79] proposed an explainable AI approach for constitutive modeling to elucidate the black box of neural networks and their high-dimensional representations, supported by a systematic hyperparameter search strategy that identifies the best neural network architectures and training parameters.

The above investigations indicate that compared with a conventional data-driven machine learning model, the predictions of which could be physically inconsistent or im-plausible, owing to the poor generalization performance caused by extrapolation or observational biases, the hybrid physics-informed and data-driven modeling method has better development prospects in terms of AI. However, since there are also limitations in the method, including the development of new algorithms and computational frameworks for diverse multi-scale and multiphysics problems, the reports presently mainly focus on the characterization of constitutive behavior. Therefore, applying hybrid physics-informed and data-driven modeling in the intelligent optimization and decision of plastic forming can be another important research direction.

## 3. Data-Driven Process Planning and Decision-Making System

### 3.1. Expert System (ES)

**Figure 12:**
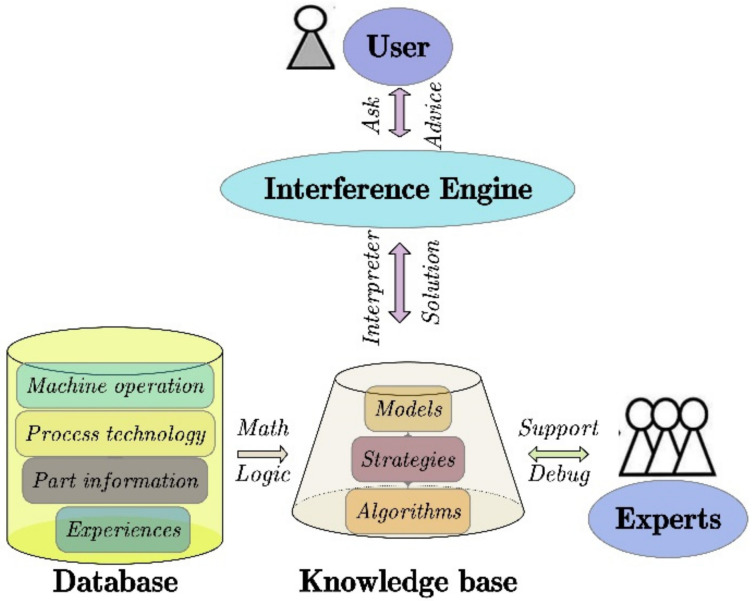
The architecture of KB-ES.

ES refers to a computer system storing a large number of specific domain knowledge. It can make inferences and draw specific suggestions according to user needs. As with human experts, it can give advice and explain the logic behind it if necessary [86]. ES is a branch of AI. It has the key characteristics of adaptive control, better processing and storage of knowledge, and reusability. It has been applied to many fields such as pattern recognition, automation, machine vision, virtual reality, automatic reasoning, data mining, and process planning [87]. The knowledge-based expert system (KB-ES) consists of three parts: database, knowledge base, and inference engine. Its overall architecture is shown in Figure 12. The database is used to store the knowledge that users are interested in. The knowledge base expresses knowledge with mathematical logic. The inference engine acts as a controlling environment and interacts with users. It receives user input about the problem, understands the knowledge base to generate inferences, and then provides expert advice.

**Figure 13:**
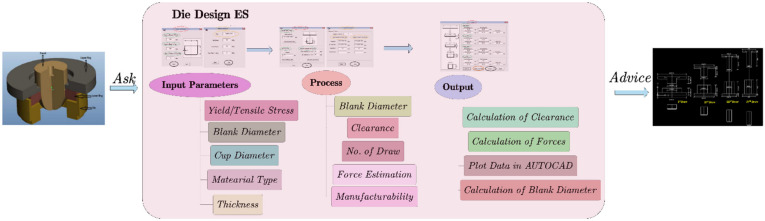
An ES of die design for multi-stage deep drawing process [90].

The computer-aided manufacturing system (CAPP) based on part feature modeling is an essential tool in the process of integrated design and manufacturing. It is also one of the widely used fields of ES. Osakada and Yang [88] utilized ANN to build an ES to guide the stamping process of rotating symmetrical parts. Due to the strong pattern recognition ability of ANN, the system can predict the most likely forming steps from the complexity of part shape, the information of die and blank materials, and the knowledge obtained from finite element simulation. The results show that the ES can reasonably predict the number of forming steps. Veera et al. [89] proposed an ES framework, which can predict the tensile properties of tailor welded blanks (TWB) according to different welding base metals and welding conditions and guide the processing process by evaluating the plastic deformation behavior of TWB. The ES has been further developed to explain more material properties, TWB conditions, material models, and other forming behaviors. Mallika and Sanjay [90] developed a set of ES for the die design of the multi-stage deep drawing process. As shown in Figure 13, the system can predict the blank diameter, blanking clearance, die contour, drawing times, punch, and blank holder force with several inputs. It is also able to generate engineering drawing for die manufacturing. The ES can directly reduce design costs and delivery time and improve productivity.

Chul Kim and Chul Woo Park [91] developed an ES for automatic process planning and die insert design for multi-mode bolt cold forging products. The system combines a program containing many expert design rules with the process variables of the commercial finite element software deform and ANSYS. This ES has four modules: input shape processing module, production feasibility inspection module, process planning module, and die design module. The suitable process is selected by reducing the number of forming sequences or the deviation distributed on the required forming load. This will minimize the tests and errors and shorten the time of developing new die sets. An ES was developed by Kim et al. [92] for the stamping forming process of 34CrMo4 steel large high-pressure vessel. The system can automatically calculate and verify the punch and die diameter of the feasible process, automatically generate the process planning diagram through the process design module, and minimize the maximum stamping load through finite element analysis, which effectively guides the stamping process design of pressure vessels. Gronostajski et al. [93] applied ES to the hot forging process. They developed an ES to evaluate the service life of forging dies, mainly to determine the impact of abrasive wear, thermomechanical fatigue, plastic deformation, and mechanical fatigue on the service life of hot forging dies. The knowledge base contains theoretical knowledge about failure phenomena, the experience of operators and industry experts, and the experience knowledge obtained from the statistical analysis of the measured data of selected forging process parameters. The knowledge is processed by automatic reasoning based on fuzzy logic rules. The results of the ES have been verified by experts, which shows that it is feasible to deal with the knowledge base using fuzzy logic rules-based inference. It also conforms to the advantage that fuzzy theory can make good use of experience. The ES is expected to analyze any forging process more completely by expanding the knowledge base module and effectively predicting the tool life of most industrial forging processes.

Previous research found that ES is usually used in the stamping process, while fewer forging ES is developed since the forging process is more complex. It is urgent to develop a universal ES to guide production in this field. ES integrates various theoretical knowledge, traditional experience, optimization algorithm, and machine learning. It carries out the preliminary design and optimization decisions for the plastic forming process, significantly improving product production efficiency and quality. Developing user-friendly KBS combined with a modern DL-based decision-making algorithm and adaptive control method is a future research direction.

### 3.2. Digital Twin (DT) System

DT is one of the most promising enabling technologies to realize intelligent manufacturing and industry 4.0. In the field of manufacturing. Garetti et al. [94] defined DT as follows: “The DT consists of a virtual representation of a production system that is able to run on different simulation disciplines that are characterized by the synchronization between the virtual and real system, thanks to sensed data and connected smart devices, mathematical models and real-time data elaboration”. The biggest feature of DT is the integration of physical space and virtual space, which can integrate physical and virtual data in the whole product life cycle. It generates a large number of data that can be analyzed and processed by intelligent algorithms and machine learning and promote the intelligence of the manufacturing industry in analysis and evaluation, predictive decision-making, and performance optimization.

**Figure 14:**
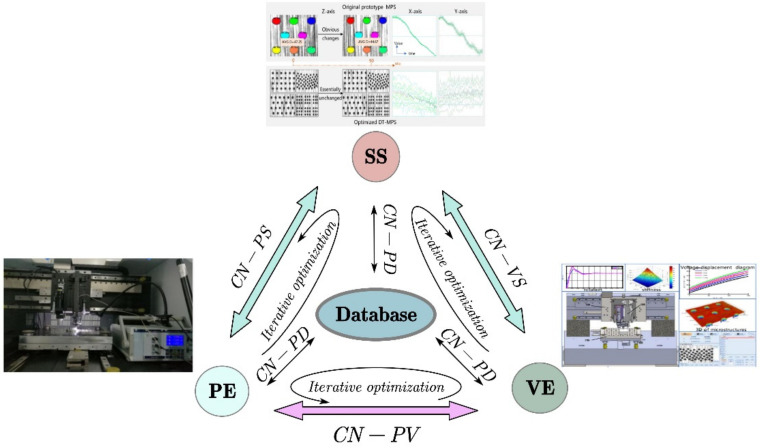
The framework of DT system [98].

Tao et al. [95] proposed that a complete DT should include five dimensions: physical part, virtual part, connection, data, and service. The framework of DT is shown in Figure 14, where PE represents the physical entity, VE represents the virtual entity, SS represents the services for both PE and VE, DD stands for the DT data, and CN means the connection of different parts. The physical part is the basis for building the virtual part. Virtual parts support the simulation, decision-making, and control of physical parts. Data are at the heart of the DT system because it is a prerequisite for creating new knowledge. In addition, DT brings new services that can enhance the convenience, reliability, and productivity of engineering systems. Finally, the connection part connects the physical part, the virtual part, data, and services [96]. DT’s industrial applications focus on product design, production, prediction, and health management (PHM). Schleich et al. [97] proposed a new DT model, which can clearly distinguish the conceptual model and its digital twin virtual representation, and used the product geometry change management as a case study. The proposed conceptual framework promoted the application of DT in the whole product life cycle.

The DT model can be applied to many automated process manufacturing systems, such as customized furniture production lines, 3C product production lines, and the plastic forming line. Based on the case study of sheet metal assembly, Söderberg et al. [99] described the functions and data models required for real-time geometric assurance in detail and how this concept can change from mass production to more personalized production. At present, the most extensive application of DT is PHM. Tuegel et al. [100] applied the DT to predict the structural life of aircraft through multi-physical modeling, multi-scale damage modeling, integration of structural finite element model (FEM) and damage model, uncertainty quantification, and high-resolution structural analysis. They reported that DT could facilitate the management of aircraft service life. Li et al. [101] established a DT model based on a dynamic Bayesian network to monitor the operation status of aircraft wings. The author established a probability model to replace the deterministic physical model. Based on the case study of the leading edge of an aircraft wing, the DT model obtains a more accurate diagnosis and prediction. Knapp et al. [102] developed a DT of the additive manufacturing process to predict cooling rate, temperature gradient, micro-hardness, velocity distribution, and solidification parameters.

**Figure 15:**
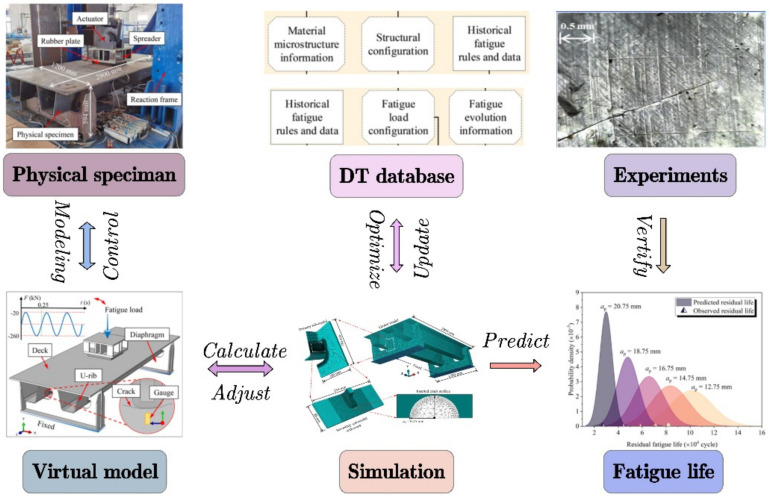
DT-driven framework for non-deterministic fatigue life prediction of steel bridges [105].

The DT-driven PHM is also highly applicable to the prediction of crack initiation. Cerrone et al. [103] proposed a DT model of the specimen based on finite element simulation and successfully solved the problem of the fuzzy crack path. The future application of DT in PHM should consider conducting more accurate multi-scale modeling based on a microstructure to simulate the crack propagation process from initiation to failure. Yeratapally et al. [104] established and demonstrated a multi-scale, uncertain DT framework for predicting fatigue crack initiation in the propagation failure of aluminum alloys. This method successfully extended the fatigue crack growth model based on a probabilistic microstructure to all probability predictions of fatigue life. Compared with the expected value of predicted fatigue life, the absolute percentage error is 9.5%. The results show that this DT-based PHM is feasible. Jiang et al. [105] developed a DT-driven framework for non-deterministic fatigue life prediction of steel bridges, as shown in Figure 15. After obtaining the critical model parameter via crystal plastic finite element simulation, the modified model was further calibrated using the assumed historical fatigue data in the DT database. The feasibility of the proposed framework was demonstrated through fatigue tests on a segmental steel deck specimen with mixed-mode deformed U-rib to diaphragm welded joints. The results show that the predicted fatigue initiation life and residual fatigue life are in good agreement with the experimentally observed life results. This indicates that DT can simulate the life cycle of physical objects at various scales, which is essential in PHM.

**Figure 16:**
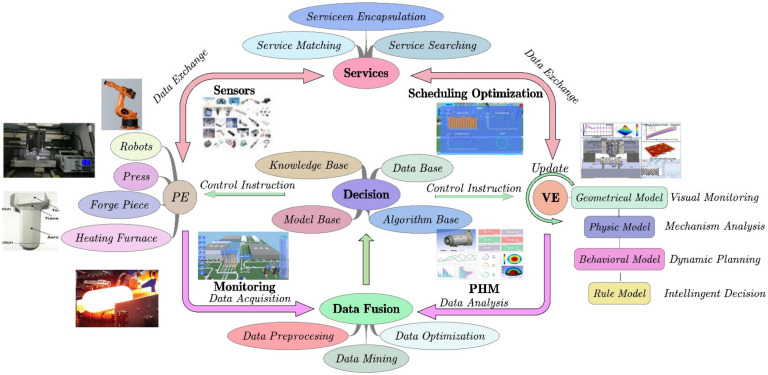
The architecture of DT system for the plastic forming process.

To date, the DT systems for plastic forming are very limited, and most focus on monitoring the production line and PHM problems. The DT-based optimization and decision for the plastic forming process, which is most important for quality control of the product, is still unavailable. The architecture of a DT system for the plastic forming process is shown in Figure 16. It consists of PE, VE, data fusion module, decision module, service module, and communication module (not explicitly plotted). The PE includes the forged piece, the relevant machine, and the whole workshop. The digital VE has four models: geometrical model, physic model, behavioral model, and rule model, which realize visual monitoring, mechanism analysis, dynamic planning, and intelligent decision making, respectively. The data fusion module processes and stores the multi-source heterogeneous data from PE and VE, and transfers the data to the decision module. The intelligent decision module consists of a database, knowledge base, model base, and algorithm base. Based on the stored data and the real-time detection data, the decision module optimizes the forming process according to previously established knowledge, model, and optimization algorithm. It then issues the control instructions to PE and VE to control the forming quality in time. The service module is a user-oriented module that efficiently provides various services for different fields and levels of users under various application scenarios. The communication module offers all the data exchange in the DT system, which guarantees the real-time interaction of reality and virtual.

In sum, DT is mainly used in PHM. It is expected to realize the online detection of part damage and the online observation of microstructure evolution by establishing a multi-scale DT model based on microstructure. DT has developed rapidly in recent years, and the relevant literature has increased. However, the development of DT is still at the conceptual stage, and its popularity in many small and medium-sized enterprises is not as good as that of an expert system. The core of DT is modeling, but there is currently no unified DT modeling method. In addition, how to filter, optimize and transmit a large amount of data are urgent problems for DT. DT has little research on plastic forming, mainly related to the high complexity of the plastic forming process itself. It can be preferentially applied to simple process and production line design optimization in the future.

## 4. Conclusions and Outlook

### 4.1. Conclusions

Considering the many advantages and potential of intelligent plastic forming, scholars have conducted a series of studies on geometric parameters, process parameters, microstructure and mechanical properties, and workshop scheduling optimization. In this paper, the research progress in the field of plastic forming was investigated. The conclusions and prospects are as follows:

- For some complex processes, the SAO method is widely used, and the optimization is gradually moving towards a diversified intelligent design with a multi-objective combination of multiple optimization algorithms and multiple optimization models. These methods complement each other and have been paid more and more attention to and applied in the field of forming process optimization design in recent years.
- The combination of RL and DL has become the current trend of optimal design due to their excellent decision-making performance and feature extraction performance. Meanwhile, the optimization design of the whole plastic forming process is changing to universality, integrity, rapidity, and user operability. The development based on ES and DT system is the outstanding performance of this characteristic.
- The DT system has shown great potential in production design and PHM. It enables the online detection of parts and the online observation of microstructure evolution by establishing a multi-scale model. However, the DT systems for plastic forming are very limited, especially for the overall process optimization and decision.

### 4.2. Outlook

Although intelligent optimization of the plastic forming process brings many conveniences, many problems also need to be addressed.

- Intelligent optimization combining machine learning and global searching algorithms lacks intrinsic mechanisms and interpretability. It is then difficult to generate universal knowledge that can be transferred to guide the forming process design of other products. It is essential to couple the physical rules into the optimization method and develop an interpretable approach to process modeling and optimization, changing the optimization process from a black box to a glass box.
- The optimization of the plastic forming process is closely related to the implicit relationship between material composition, process, microstructure, and properties. All the optimization design of material composition and process schemes aiming at performance must be closely combined with microstructures to truly grasp the essential law of process optimization. This means that the objective of design optimization has changed from macro parameters to macro micro coupling parameters, the dimension of design optimization has increased sharply, the response of the objective is highly nonlinear, and the difficulty of modeling and calculation has increased greatly. Therefore, with the increasing demand for intelligent optimization design of plastic forming, the field of multi-scale design optimization urgently needs to be studied.
- With the development of plastic forming technology, the contradiction between the accuracy and efficiency of design optimization has become increasingly prominent. Various results can be obtained in terms of efficiency and accuracy using different optimization methods, combined with diverse sample selection, numerical simulation, and optimization algorithms. In order to achieve an efficient optimization design of the forming process, it is urgent to develop alternative models for simplifying sequential modeling and large-scale parallel computing.
- Although some knowledge-based automatic design optimization methods and integrated computing platforms have been developed to integrate sample selection, numerical modeling, and optimization algorithms, most methods are still based on manual operation and model-driven optimization. There are substantial data, rules, and various knowledge in the forming production, which is far from meeting the urgent requirements for online real-time design optimization. Therefore, there is an urgent need to develop general expert systems based on emerging AI technologies such as big data, cloud computing, and machine learning to promote the development of digital twins, from model-driven to data-driven [106,107,108].
- The critical technology for the DT system is still at a premature stage. To establish a high-fidelity digital model of workpiece and forming equipment, and realize the real-time control of PE and VE, the following key technologies should be developed: (1) intelligent detection accompanied by the fast intelligent reconstruction of macro and micro physical field (i.e., temperature, stress field, grain size, mechanical property, etc.); (2) data fusion technology for multi-source and heterogenous data; (3) fieldbus technology for the real-time communication between each module of DT system; (4) a hybrid prediction model driven by data and intrinsic mechanism multi-filed for accurate and fast simulation of multi-field and whole process simulation of plastic forming.

## Figures and Tables

**Table 1:** A summary of intelligent optimization methods for stamping.

Method	Dataset	Optimization Parameters	Input Parameters	R^2^	Reference
MOGA + RSM	15	Fracture, wrinkle, insufficient stretching and thickness	BHF, draw-bead restraining force	/	[17]
GA + ANN	50	BHF curve	Die parameters	0.949	[37]
Bayesian	13	Four springback angles of the specimen	Tool radius, BHF, sheet thickness	0.965	[23]
MOPSO + RBM-ANN	40	Maximum thinning rate, per- centage thickening area	BHF, die parameters	0.966	[22]
MCS + RSM	57	Wrinkle, maximum thinning rate	BHF, sheet and die parameters,	0.981	[25]
	15	The maximum thinning percentage, springback measure	BHF, lubricating conditions, strain-hardening index of the material	0.987	[26]
NSGA + ANN ^2^	70	Springback measure	Material type, process parameters	0.98	[38]
PSO + ANN	36	Sewall angle, flange angle, sidewall curvature	BHF, punch velocity, die-blank, etc.	0.98	[39]
SSA + ANN ^1^	160	Maximum springback, springback radius, thickness of the sheet	Sheet type, punch size, bending radius, etc.	0.96	[40]
CNN	1800	Full thinning and displacement fields	Die geometry, blank geometry, spacer thickness, etc.	/	[27]

^1^ Sparrow search algorithm (SSA). ^2^ Non-dominated sorting genetic algorithm (SSA).

**Table 2:** A summary of intelligent optimization methods for forging.

Method	Dataset	Optimization Parameters	Input Parameters	R^2^	References
GA + ANN	40	Forging force and die stress	Preform geometry parameters	0.95	[42]
	10	Forging force	Die geometry parameters	/	[44]
	25	Forging load, energy absorbed	Billet dimensions	0.969	[54]
NSGA-II + RSM	46	Maximum filling ratio of the final die, minimum flash volume, etc.	Preform geometry parameters	0.954	[43]
	25	Deformation homogeneity and material damage	Billet rotating speed, hammer radial feed rate, etc.	0.99	[55]
Weighted LP norm + RBF ^1^	15	Risk of material damage, forging energy	Initial load, stiffness	/	[45]
ANN	600	Grain size	Initial temperature, transport time, pause time, strain rate	0.998	[19]
GRA + Taguchi ^2^	27	Forging load and billet temperature loss	Flash thickness, die temperature, etc.	0.935	[56]
NSGA-II + ANN	/	Uniformity of strain distribution, flash volume, lateral forces	Geometrical dimensions of preform shape, etc.	0.82	[57]
CNN	240	Forging force	Preform geometry parameters	0.989	[18]

^1^ Radial basis function network (RBF). ^2^ Grey relational analysis (GRA).

**Table 3:** Comparison of prediction accuracy of different machine learning algorithms [20].

Algorithm	MSE	R^2^
SVM (poly)	7731.26	0.4(±0.02)
SVM (rbf)	7769.838	0.465(±0.02)
KNN	721.241	0.827(±0.01)
Linear regression	765.9897	0.85(±0.01)
Random forest	623.4395	0.896(±0.01)
DNN	553.258	0.907(±0.01)

**Table 4:** A summary of intelligent optimization methods for rolling.

Method	Dataset	Optimization Parameters	Input Parameters	R^2^/MSE	References
GA + ANN	1440	Bending force	Entrance temperature and thickness, etc	0.983	[62]
	188	Flatness value	Entrance temperature and thickness, etc	0.79	[63]
PSO + ELM ELM PSO + SVM	490	Roll force and roll torque	Rolling reduction rate, roll radius, rolling speed, etc.	0.9999 0.9991 0.9691	[58]
ANN NSGA II-ANN DNN	10,133	Strip crown	Entrance temperature and thickness etc.	0.9899 0.9903 0.9910	[59]
MCMC + Bayesian ^1^	5000	Quality attributes, microstructural features.	Roll Loads, temperature, speeds, etc.	0.95	[60]
GA + GLR ^2^	1994	Width deviation	Entry surface temperature, relative reduction of thickness, etc.	0.0177	[61]

^1^ Markov Chain Monte Carlo (MCMC); ^2^ Generalized linear regression (GLR).

**Table 5:** A summary of intelligent optimization methods for spinning.

Method	Dataset	Optimization Parameters	Input Parameters	R^2^/MSE	References
SDM + ANN ^1^	48	Dimensions of the formed part	Tool-path parameters, sizes of a blank disk of sheet metal, tool, die	0.90	[21]
PSO + GRP PSO + SVM PSO + DNN	16	Wall thickness reduction and flange fluctuation degree	Inner radius of flange, width of flange, etc.	0.64% 1.67% 0.17%	[66]
PSO + ANN	64	Mean thickness	Axial stagger, feed speed ratio, etc.	97.67%	[67]

^1^ Steepest descent method (SDM).

## Data Availability

Not applicable.

## References

[1] Ngaile G., Kinsey B. (2011). Advances in Plastic Forming of Metals. J. Manuf. Sci. Eng..

[2] Lee M.-G., Korkolis Y. (2018). Advances in Plastic Forming of Metals. Metals.

[3] Jiang P., He X.M., Yang Y., Zhou L.Y. (2020). Development of domestic precision plastic forming technology and its application in industrial production. Die Mould Ind..

[4] Li H., Yang J., Chen G., Liu X., Zhang Z., Li G., Liu W. (2021). Towards intelligent design optimization: Progress and challenge of design optimization theories and technologies for plastic forming. Chin. J. Aeronaut..

[5] Liu R.J., Zhang Y.W., Wen C.W., Tang J. (2010). Study on the design and analysis methods of orthogonal experiment. Exp. Technol. Manag..

[6] Chen C.T., Gu G.X. (2020). Generative Deep Neural Networks for Inverse Materials Design Using Backpropagation and Active Learning. Adv. Sci..

[7] Tagade P.M., Adiga S.P., Pandian S., Park M.S., Hariharan K.S., Kolake S.M. (2019). Attribute driven inverse materials design using deep learning Bayesian framework. NPJ Comput. Mater..

[8] Zhou Q., Lu S., Wu Y., Wang J. (2020). Property-Oriented Material Design Based on a Data-Driven Machine Learning Technique. J. Phys. Chem. Lett..

[9] Senthilkumar K.M., Thirumalai R., Selvam T.A., Natarajan A., Ganesan T. (2021). Multi objective optimization in machining of Inconel 718 using taguchi method. Mater. Today Proc..

[10] Thirumalai R., Seenivasan M., Panneerselvam K. (2021). Experimental investigation and multi response optimization of turning process parameters for Inconel 718 using TOPSIS approach. Mater. Today Proc..

[11] Thirumalai R., Techato K., Chandrasekaran M., Venkatapathy K., Seenivasan M. (2021). Experimental investigation during turning process of titanium material for surface roughness. Mater. Today Proc..

[12] Joshi S.N., Pande S.S. (2011). Intelligent process modeling and optimization of die-sinking electric discharge machining. Appl. Soft Comput..

[13] Wang X., Yan Y., Gu X. (2019). Spot welding robot path planning using intelligent algorithm. J. Manuf. Process..

[14] Choudhary A., Kumar M., Gupta M.K., Unune D.K., Mia M. (2019). Mathematical modeling and intelligent optimization of submerged arc welding process parameters using hybrid PSO-GA evolutionary algorithms. Neural Comput. Appl..

[15] Medhi T., Hussain S.A.I., Roy B.S., Saha S.C. (2021). An intelligent multi-objective framework for optimizing friction-stir welding process parameters. Appl. Soft Comput..

[16] Yifei T., Meng Z., Jingwei L., Dongbo L., Yulin W. (2018). Research on Intelligent Welding Robot Path Optimization Based on GA and PSO Algorithms. IEEE Access.

[17] Wei L., Yuying Y. (2008). Multi-objective optimization of sheet metal forming process using Pareto-based genetic algorithm. J. Mater. Process. Technol..

[18] Lee S., Quagliato L., Park D., Kwon I., Sun J., Kim N. (2021). A New Approach to Preform Design in Metal Forging Processes Based on the Convolution Neural Network. Appl. Sci..

[19] Bambach M., Imran M., Sizova I., Buhl J., Gerster S., Herty M. (2021). A soft sensor for property control in multi-stage hot forming based on a level set formulation of grain size evolution and machine learning. Adv. Ind. Manuf. Eng..

[20] Xie Q., Suvarna M., Li J., Zhu X., Cai J., Wang X. (2021). Online prediction of mechanical properties of hot rolled steel plate using machine learning. Mater. Des..

[21] Gondo S., Arai H. (2022). Data-driven metal spinning using neural network for obtaining desired dimensions of formed cup. CIRP Ann..

[22] Xie Y., Du L., Zhao J., Liu C., Li W. (2021). Multi-objective optimization of process parameters in stamping based on an improved RBM–BPNN network and MOPSO algorithm. Struct. Multidiscip. Optim..

[23] Spathopoulos S., Stavroulakis G. (2020). Springback Prediction in Sheet Metal Forming, Based on Finite Element Analysis and Artificial Neural Network Approach. Appl. Mech..

[24] Cai H., Xiao W., Zheng K. (2022). The prediction of part thickness using machine learning in aluminum hot stamping process with partition temperature control. Int. J. Adv. Manuf. Technol..

[25] Gantar G., Kuzman K. (2005). Optimization of stamping processes aiming at maximal process stability. J. Mater. Process. Technol..

[26] Marretta L., Ingarao G., Di Lorenzo R. (2010). Design of sheet stamping operations to control springback and thinning: A multi-objective stochastic optimization approach. Int. J. Mech. Sci..

[27] Attar H.R., Zhou H., Foster A., Li N. (2021). Rapid feasibility assessment of components to be formed through hot stamping: A deep learning approach. J. Manuf. Process..

[28] Zhou H., Xu Q., Nie Z., Li N. (2021). A Study on Using Image-Based Machine Learning Methods to Develop Surrogate Models of Stamp Forming Simulations. J. Manuf. Sci. Eng..

[29] Chokshi P., Dashwood R., Hughes D.J. (2017). Artificial Neural Network (ANN) based microstructural prediction model for 22MnB5 boron steel during tailored hot stamping. Comput. Struct..

[30] Hu F.K., Zhu Z.J., Wang K., Zhu B., Zhang Y.S. (2019). Identification of hot stamping fully martenstic microstructure SEM photograph with support vector machine. Advanced High Strength Steel and Press Hardening, Proceedings of the 4th International Conference on Advanced High Strength Steel and Press Hardening, Hefei, China, 20–22 August 2018.

[31] Hart-Rawung T., Buhl J., Bambach M. (2020). A Fast Approach for Optimization of Hot Stamping Based on Machine Learning of Phase Transformation Kinetics. Procedia Manuf..

[32] Cheng M., Fang F., Pain C.C., Navon I.M. (2020). Data-driven modelling of nonlinear spatio-temporal fluid flows using a deep convolutional generative adversarial network. Comput. Methods Appl. Mech. Eng..

[33] Hao P., Liu D., Zhang K., Yuan Y., Wang B., Li G., Zhang X. (2021). Intelligent layout design of curvilinearly stiffened panels via deep learning-based method. Mater. Des..

[34] Liu S., Shi Z., Lin J., Li Z. (2020). Reinforcement learning in free-form stamping of sheet-metals. Procedia Manuf..

[35] Zimmerling C., Poppe C., Stein O., Kärger L. (2022). Optimisation of manufacturing process parameters for variable component geometries using reinforcement learning. Mater. Des..

[36] Lockner Y., Hopmann C. (2021). Induced network-based transfer learning in injection molding for process modelling and optimization with artificial neural networks. Int. J. Adv. Manuf. Technol..

[37] Zhang H.W., Zheng X.T. (2020). Blank Holder Force Prediction of Tailor Welded Blank Based on Neural Network Optimized by Genetic. J. Northeast. Univ. (Nat. Sci.).

[38] Liew K.M., Ray T., Tan H., Tan M.J. (2002). Evolutionary Optimization and Use of Neural Network for Optimum Stamping Process Design for Minimum Springback. J. Comput. Inf. Sci. Eng..

[39] El Mrabti I., Touache A., El Hakimi A., Chamat A. (2021). Springback optimization of deep drawing process based on FEM-ANN-PSO strategy. Struct. Multidiscip. Optim..

[40] Li L., Zhang Z., Xu B. (2022). Prediction of Spherical Sheet Springback Based on a Sparrow-Search-Algorithm-Optimized BP Neural Network. Metals.

[41] Li J.J., Huang M.l., Peng Q.Z. (2015). Development Status and Trends of Forging Technology. Heat Treat. Technol. Equip..

[42] Zhang M.Y., Wang X.Y., Xia J.C., Ji G. (2010). Multiple-target optimization design of pre-forging for gear blank using back propagation neural network and genetic algorithm. Forg. Stamp. Technol..

[43] Torabi S.H.R., Alibabaei S., Bonab B.B., Sadeghi M.H., Faraji G. (2015). Design and optimization of turbine blade preform forging using RSM and NSGA II. J. Intell. Manuf..

[44] Wu Y.J., Zhao Z., Liang Y.Q., Hu C.L., Gao C.H. (2009). Optimization of preform of high-speed multi-stage forging based on surrogate model methodology. J. Plast. Eng..

[45] Kitayama S., Kadoya S., Takano M., Kobayashi A. (2021). Multi-objective optimization of process parameters in cold forging minimizing risk of crack and forging energy. Arch. Civ. Mech. Eng..

[46] Hu F., Wu Z.P., Wang D.H., Zhang W.H. (2017). Sequential approximate optimization method. J. Natl. Univ. Def. Technol..

[47] Xu F., Dong X.H., Wang X.B., Li Z.D., Liu Q. (2019). Optimization on radial forging process of rectangular cross-section billet. Forg. Stamp. Technol..

[48] Wang J.M. (2004). Research on Optimization Decision Theory and Application of Multi-Objective Fuzzy Recognition. Ph.D. Thesis.

[49] Mori T., Uchihira N. (2019). Balancing the trade-off between accuracy and interpretability in software defect prediction. Empir. Softw. Eng..

[50] Chen M.-S., Yuan W.-Q., Li H.-B., Zou Z.-H. (2018). New insights on the relationship between flow stress softening and dynamic recrystallization behavior of magnesium alloy AZ31B. Mater. Charact..

[51] Shu D., Wang J., Jiang M., Chen G., Lu L., Zhang H. (2021). Modeling of Dynamic Recrystallization Behavior of As-Extruded AM50 Magnesium Alloy during Hot Compression by a Cellular Automaton Method. Metals.

[52] Chen D.-D., Lin Y.-C., Chen X.-M. (2019). A strategy to control microstructures of a Ni-based superalloy during hot forging based on particle swarm optimization algorithm. Adv. Manuf..

[53] Ponnusami S.A. (2021). From microstructural images to properties—An interpretable deep learning approach to predict elastic-plastic properties of fiber composites.

[54] Ciancio C., Citrea T., Ambrogio G., Filice L., Musmanno R. (2015). Design of a High Performance Predictive Tool for Forging Operation. Procedia CIRP.

[55] Zhu F., Wang Z., Lv M. (2016). Multi-objective optimization method of precision forging process parameters to control the forming quality. Int. J. Adv. Manuf. Technol..

[56] Equbal M.I., Kumar R., Shamim M., Ohdar R.K. (2014). A Grey-based Taguchi Method to Optimize Hot Forging Process. Procedia Mater. Sci..

[57] Alimirzaloo V., Biglari F.R., Sadeghi M.H., Keshtiban P.M., Sehat H.R. (2019). A novel method for preform die design in forging process of an airfoil blade based on Lagrange interpolation and meta-heuristic algorithm. Int. J. Adv. Manuf. Technol..

[58] Wang Z., Zhang D., Gong D., Peng W. (2019). A New Data-driven Roll Force and Roll Torque Model Based on FEM and Hybrid PSO-ELM for Hot Strip Rolling. ISIJ Int..

[59] Deng J., Sun J., Peng W., Hu Y., Zhang D. (2019). Application of neural networks for predicting hot-rolled strip crown. Appl. Soft Comput..

[60] Agarwal K., Shivpuri R. (2012). An On-Line Hierarchical Decomposition Based Bayesian Model for Quality Prediction during Hot Strip Rolling. ISIJ Int..

[61] Ji Y., Liu S., Zhou M., Zhao Z., Guo X., Qi L. (2022). A machine learning and genetic algorithm-based method for predicting width deviation of hot-rolled strip in steel production systems. Inf. Sci..

[62] Wang Z.-H., Gong D.-Y., Li X., Li G.-T., Zhang D.-H. (2017). Prediction of bending force in the hot strip rolling process using artificial neural network and genetic algorithm (ANN-GA). Int. J. Adv. Manuf. Technol..

[63] John S., Sikdar S., Swamy P.K., Das S., Maity B. (2008). Hybrid neural–GA model to predict and minimise flatness value of hot rolled strips. J. Mater. Process. Technol..

[64] Xia Q., Xiao G., Long H., Cheng X., Sheng X. (2014). A review of process advancement of novel metal spinning. Int. J. Mach. Tools Manuf..

[65] Gao P.F., Yan X.G., Li F.G., Zhan M., Ma F., Fu M.W. (2022). Deformation mode and wall thickness variation in conventional spinning of metal sheets. Int. J. Mach. Tools Manuf..

[66] Gao P., Yan X., Wang Y., Li H., Zhan M., Ma F., Fu M. (2022). An online intelligent method for roller path design in conventional spinning. J. Intell. Manuf..

[67] Banerjee P., Hui N.B., Dikshit M.K., Laha R., Das S. (2020). Modelling and optimization of mean thickness of backward flow formed tubes using regression analysis, particle swarm optimization and neural network. SN Appl. Sci..

[68] Wang X.H., Zhang L., Ren L., Xie K.G., Wang K.Y., Ye F., Chen Z. (2021). Brief Review on Applying Reinforcement Learning to Job Shop Scheduling Problems. J. Syst. Simul..

[69] Hartmanis J. (1982). Computers and Intractability: A Guide to the Theory of NP-Completeness (Michael R. Garey and David S. Johnson). Siam Rev..

[70] Çaliş B., Bulkan S. (2015). A research survey: Review of AI solution strategies of job shop scheduling problem. J. Intell. Manuf..

[71] Chaudhry I.A., Khan A. (2016). A Research Survey: Review of Flexible Job Shop Scheduling Techniques. Int. Trans. Oper. Res..

[72] Seng D., Li J., Fang X., Zhang X., Chen J. (2018). Low-Carbon Flexible Job-Shop Scheduling Based on Improved Nondominated Sorting Genetic Algorithm-II. Int. J. Simul. Model..

[73] Wang H., Sheng B., Lu Q., Yin X., Zhao F., Lu X., Luo R., Fu G. (2021). A novel multi-objective optimization algorithm for the integrated scheduling of flexible job shops considering preventive maintenance activities and transportation processes. Soft Comput..

[74] Mnih V., Kavukcuoglu K., Silver D., Graves A., Antonoglou I., Wierstra D., Riedmiller M.A. (2013). Playing Atari with Deep Reinforcement Learning. arXiv.

[75] Nazari M., Oroojlooy A., Takáč M., Snyder L.V. Reinforcement learning for solving the vehicle routing problem. Proceedings of the 32nd International Conference on Neural Information Processing Systems.

[76] Bello I., Pham H., Le Q., Norouzi M., Bengio S. (2016). Neural Combinatorial Optimization with Reinforcement Learning. arXiv.

[77] Zhang C., Song W., Cao Z., Zhang J., Tan P., Xu C. (2020). Learning to Dispatch for Job Shop Scheduling via Deep Reinforcement Learning. Adv. Neural Inf. Process. Syst..

[78] Luo Z.H., Jiang C.L., Liu L., Zheng X.L., Ma H.D. (2022). Research on deep reinforcement learning based intelligent shop scheduling method. Chin. J. Internet Things.

[79] Luo S. (2020). Dynamic scheduling for flexible job shop with new job insertions by deep reinforcement learning. Appl. Soft Comput..

[80] Lv S.Y., Yu P. (2022). A Review of Green Flexible Job-Shop Scheduling Problem. Logist. Eng. Manag..

[81] Zhou X.H. (2022). Research on the Integration of Artificial Intelligence, Big Data and Cloud Computing. Comput. Knowl. Technol..

[82] Gui X.Y., Li S.A. (2022). Computer Network Cloud Computing Technology. Yangtze River Inf. Commun..

[83] Wang A., Zheng Y., Liu J., Fakir O.E., Masen M.A., Wang L. (2016). Knowledge Based Cloud FE simulation—Data-driven material characterization guidelines for the hot stamping of aluminium alloys. J. Phys. Conf. Ser..

[84] Luan X., Zhang Q., Elfakir O., Wang L., Gharbi M.M. (2017). Uni-Form: A Pilot Production Line for Hot/Warm Sheet Metal Forming Integrated in a Cloud Based SMARTFORMING Platform. Advanced High Strength Steel and Press Hardening: Proceedings of the 3rd International Conference on Advanced High Strength Steel and Press Hardening (ICHSU2016), Xi’an, China, 25–27 August 2016.

[85] Wang A., El Fakir O., Liu J., Zhang Q., Zheng Y., Wang L. (2019). Multi-objective finite element simulations of a sheet metal-forming process via a cloud-based platform. Int. J. Adv. Manuf. Technol..

[86] Shu-Hsien L. (2005). Expert system methodologies and applications—A decade review from 1995 to 2004. Expert Syst. Appl..

[87] Yusof Y., Latif K. (2014). Survey on computer-aided process planning. Int. J. Adv. Manuf. Technol..

[88] Osakada K., Yang G. (1991). Application of neural networks to an expert system for cold forging. Int. J. Mach. Tools Manuf..

[89] Veera Babu K., Ganesh Narayanan R., Saravana Kumar G. (2009). An expert system based on artificial neural network for predicting the tensile behavior of tailor welded blanks. Expert Syst. Appl..

[90] Bhatt M.R., Buch S.H. (2017). An Expert System of Die Design for Multi Stage Deep Drawing Process. Procedia Eng..

[91] Kim C., Park C.W. (2006). Development of an expert system for cold forging of axisymmetric product. Int. J. Adv. Manuf. Technol..

[92] Kim C.H., Park J.H., Kim C., Choi J.C. (2004). Expert system for process planning of pressure vessel fabrication by deep drawing and ironing. J. Mater. Process. Technol..

[93] Gronostajski Z., Hawryluk M., Kaszuba M., Marciniak M., Niechajowicz A., Polak S., Zwierzchwoski M., Adrian A., Mrzygłód B., Durak J. (2016). The expert system supporting the assessment of the durability of forging tools. Int. J. Adv. Manuf. Technol..

[94] Garetti M., Rosa P., Terzi S. (2012). Life Cycle Simulation for the design of Product-Service Systems. Comput. Ind..

[95] Tao F., Zhang M. (2017). Digital Twin Shop-Floor: A New Shop-Floor Paradigm Towards Smart Manufacturing. IEEE Access.

[96] Tao F., Zhang H., Liu A., Nee A.Y.C. (2019). Digital Twin in Industry: State-of-the-Art. IEEE Trans. Ind. Inform..

[97] Schleich B., Anwer N., Mathieu L., Wartzack S. (2017). Shaping the digital twin for design and production engineering. CIRP Ann..

[98] Tao F., Zhang M., Liu Y., Nee A.Y.C. (2018). Digital twin driven prognostics and health management for complex equipment. CIRP Ann..

[99] Söderberg R., Wärmefjord K., Carlson J.S., Lindkvist L. (2017). Toward a Digital Twin for real-time geometry assurance in individualized production. CIRP Ann..

[100] Tuegel E.J., Ingraffea A.R., Eason T.G., Spottswood S.M. (2011). Reengineering Aircraft Structural Life Prediction Using a Digital Twin. Int. J. Aerosp. Eng..

[101] Li C., Mahadevan S., Ling Y., Choze S., Wang L. (2017). Dynamic Bayesian Network for Aircraft Wing Health Monitoring Digital Twin. AIAA J..

[102] Knapp G.L., Mukherjee T., Zuback J.S., Wei H.L., Palmer T.A., De A., DebRoy T. (2017). Building blocks for a digital twin of additive manufacturing. Acta Mater..

[103] Cerrone A., Hochhalter J., Heber G., Ingraffea A. (2014). On the Effects of Modeling As-Manufactured Geometry: Toward Digital Twin. Int. J. Aerosp. Eng..

[104] Yeratapally S.R., Leser P.E., Hochhalter J.D., Leser W.P., Ruggles T.J. (2020). A digital twin feasibility study (Part I): Non-deterministic predictions of fatigue life in aluminum alloy 7075-T651 using a microstructure-based multi-scale model. Eng. Fract. Mech..

[105] Jiang F., Ding Y., Song Y., Geng F., Wang Z. (2021). Digital Twin-driven framework for fatigue life prediction of steel bridges using a probabilistic multiscale model: Application to segmental orthotropic steel deck specimen. Eng. Struct..

[106] Jeschke S., Brecher C., Meisen T., Özdemir D., Eschert T., Jeschke S., Brecher C., Song H., Rawat D.B. (2017). Industrial Internet of Things and Cyber Manufacturing Systems. Industrial Internet of Things: Cybermanufacturing Systems.

[107] Thoben K.-D., Wiesner S., Wuest T. (2017). “Industrie 4.0” and Smart Manufacturing—A Review of Research Issues and Application Examples. Int. J. Autom. Technol..

[108] Zheng P., Wang H., Sang Z., Zhong R.Y., Liu Y., Liu C., Mubarok K., Yu S., Xu X. (2018). Smart manufacturing systems for Industry 4.0: Conceptual framework, scenarios, and future perspectives. Front. Mech. Eng..

